# Development of Adult Renal Tubular Organoids from Different Human Individuals in a Single Medium

**DOI:** 10.31662/jmaj.2024-0244

**Published:** 2024-12-20

**Authors:** Makiko Mori, Yutaro Mori, Yuki Nakao, Shintaro Mandai, Tamami Fujiki, Hiroaki Kikuchi, Fumiaki Ando, Koichiro Susa, Takayasu Mori, Yuma Waseda, Soichiro Yoshida, Yasuhisa Fujii, Eisei Sohara, Shinichi Uchida

**Affiliations:** 1 Department of Nephrology, Graduate School of Medical and Dental Sciences, Tokyo Medical and Dental University, Tokyo, Japan; 2 Department of Urology, Graduate School of Medical and Dental Sciences, Tokyo Medical and Dental University, Tokyo, Japan

**Keywords:** Organoids, Tubuloids, Pathological model, Chronic kidney disease

## Abstract

**Introduction:**

Organoids are miniature organs developed through technology. Kidney organoids that originate from human inducible pluripotent stem cells (iPSCs) were developed to recreate renal diseases. However, it is impossible to simultaneously produce kidney organoids from iPSCs of multiple individuals and in a single medium. We herein report the development of adult renal tubular organoids, namely, “tubuloids,” from primary renal epithelial cells from multiple human individuals in a single medium.

**Methods:**

Kidneys from eight patients who underwent nephrectomy due to malignancy were sectioned, and primary renal epithelial tubule cells were cultured; four had normal kidney function, and four had mild chronic kidney disease (CKD). Growth factors and Matrigel were added to the primary culture.

**Results:**

Primary cultured renal epithelial cells from normal kidneys exhibited a fine and swollen epithelial appearance, whereas those from kidneys with mild CKD were smaller and slightly elongated. Growth was faster in normal kidney cells than in mild CKD cells. At the beginning of the three-dimensionalization (day 0), normal renal tubuloids grew faster than mild CKD tubuloids. The difference in size between normal and mild CKD tubuloids was not obvious by day 5. Both tubuloid types had comparable sizes by day 21.

**Conclusions:**

Renal tubular organoids can be developed simultaneously and in a single medium. Our method is expected to be used as a human pathological model.

## Introduction

Biomedical engineering has experienced remarkable advancements. Various diseases have been explored and elucidated using organoids, which are miniature organ-like structures that serve as an alternative to a human model ^(1), (2)^. Chronic kidney disease (CKD) is an irreversible and progressive disease characterized by glomerular sclerosis, tubular atrophy and collapse, and fibrosis and the infiltration of inflammatory cells ^(3)^. Despite its prevalence, the pathogenesis of CKD remains unclear, and a definitive treatment is lacking.

One reason for the incremental progress in CKD research may be that kidney disease is largely influenced by aging ^(4)^. Rats and mice are primarily used to understand kidney diseases; however, they cannot accurately represent its status in human organs that are >80 years old. Kidney organoids developed from human inducible pluripotent stem cells (iPSCs) are considered as a novel pathological model ^(5), (6)^. However, currently developed kidney organoids from iPSCs cannot simulate kidney function past the embryonic stage ^(7)^. Another challenge with iPSC kidney organoids is the technique required to develop them and that they cannot be created from different individuals in a uniform condition.

To develop iPSC kidney organoids, nephron progenitor cells (NPCs) should first be established from iPSCs ^(5), (6)^. Culture conditions for inducing differentiation into NPCs greatly vary depending on the origin of the iPSCs. Furthermore, NPCs do not necessarily become kidney organoids under uniform differentiation induction conditions on different strains. Thus, the inability of iPSCs from different individuals to develop kidney organoids in a uniform condition is a critical limitation for studying CKD due to various causes and factors, such as age, race, sex, and progression. This limitation makes it almost impossible to accurately analyze CKD and its response to drugs. To the best of our knowledge, iPSC kidney organoids developed from multiple human-derived iPSCs in a uniform condition have not yet been reported. In addition, mouse models simulating CKD-like diseases have not succeeded in reproducing the diversity among humans.

CKD is caused by renal tubular dysfunction more than glomerular dysfunction ^(8), (9)^. We previously focused on renal proximal tubules and successfully developed tubular organoids derived from primary human renal proximal tubular epithelial cells (hRPTECs) ^(10), (11), (12)^. In this study, we developed human adult renal tubular organoids, namely, “tubuloids,” from different individuals simultaneously and in a uniform condition. Our strategy can provide insights for analyzing the pathophysiology of kidney diseases, including CKD, in a personalized or semipersonalized fashion.

## Materials and Methods

### Cell culture experiments

Samples were obtained from kidneys surgically removed from patients with renal cell carcinoma or urothelial malignancies at Tokyo Medical and Dental University Hospital. The protocol was approved by the Institutional Review Board of the Ethics Committee of Tokyo Medical and Dental University (M2022-005). Primary hRPTECs were cultured from the areas of the kidney not infiltrated by malignancy.

First, the kidney cortex was sectioned and dissolved in collagenase type II (1.0 mg/mL) (Worthington Biochemical, NJ, USA). The cells from the digested kidney were then cultured in hRPTEC media (DMEM/F-12 (Nacalai Tesque, Kyoto, Japan) containing bovine serum albumin (Nacalai Tesque, Kyoto, Japan), antibiotic-antifungal (ThermoFisher Scientific, MA, USA), hydrocortisone (ThermoFisher Scientific, MA, USA), ITS liquid media supplement (Sigma-Aldrich, MO, USA), and human recombinant epidermal growth factor (EGF) (ThermoFisher Scientific, MA, USA). The cell culture was then incubated in a CO_2_ incubator (5% CO_2_) at 37°C for 5-7 days.

### Human renal tubuloids

hRPTECs were seeded in ultralow adhesion plates using Advanced Roswell Park Memorial Institute media (RPMI) 1640 medium with 5% fetal bovine serum (FBS, SERANA, Brandenburg, Germany). After 2 days, Matrigel (Corning, NY, USA) was added followed by Advanced RPMI with 5% FBS plus EGF, fibroblast growth factor (FGF) (ThermoFisher Scientific, MA, USA), and hepatocyte growth factor (HGF) (ThermoFisher Scientific, MA, USA). We defined the media containing growth factors as “tubuloid media.” The medium was incubated for 1-2 weeks in a CO_2_ incubator (5% CO_2_) at 37°C. The medium was changed twice a week. No other work was required; that is, the medium was left in place.

### Microscopic observation

We obtained images of all cells and tubuloids using BZ-X800 (Keyence, Osaka, Japan).

## Results

### Establishment of primary hRPTECs from resected kidneys

Table 1 shows the detailed characteristics of the eight patients who underwent nephrectomy due to malignancies. Of the eight patients, four had normal kidney function (eGFR > 60 mL/min/1.73 m^2^, named N-1–N-4), and four had mild CKD (eGFR 45-60 mL/min/1.73 m^2^, named C-1–C-4). We established the eight strains of primary hRPTECs in a two-dimensional culture condition. Notably, a decreased kidney function did not affect the efficiency of primary culture establishment.

**Table 1.** Characteristics of the Study Participants.

Nunber	Sex	Age	Cre (mg/dL)	eGFR (mL/min/1.73m2)	BUN (mg/dL)
N-1	Male	70s	0.79	72.4	9.8
N-2	Male	40s	0.62	107.1	7.9
N-3	Famale	50s	0.77	61.7	12.9
N-4	Famale	80s	0.64	66	21.3
C-1	Famale	50s	0.92	49.5	12.6
C-2	Male	60s	1.2	48.8	14.6
C-3	Male	60s	1.17	50.5	24.1
C-4	Famale	50s	0.92	49.2	14.6

### Primary hRPTECs from patients with mild CKD had an elongated morphology

**Figure 1.**
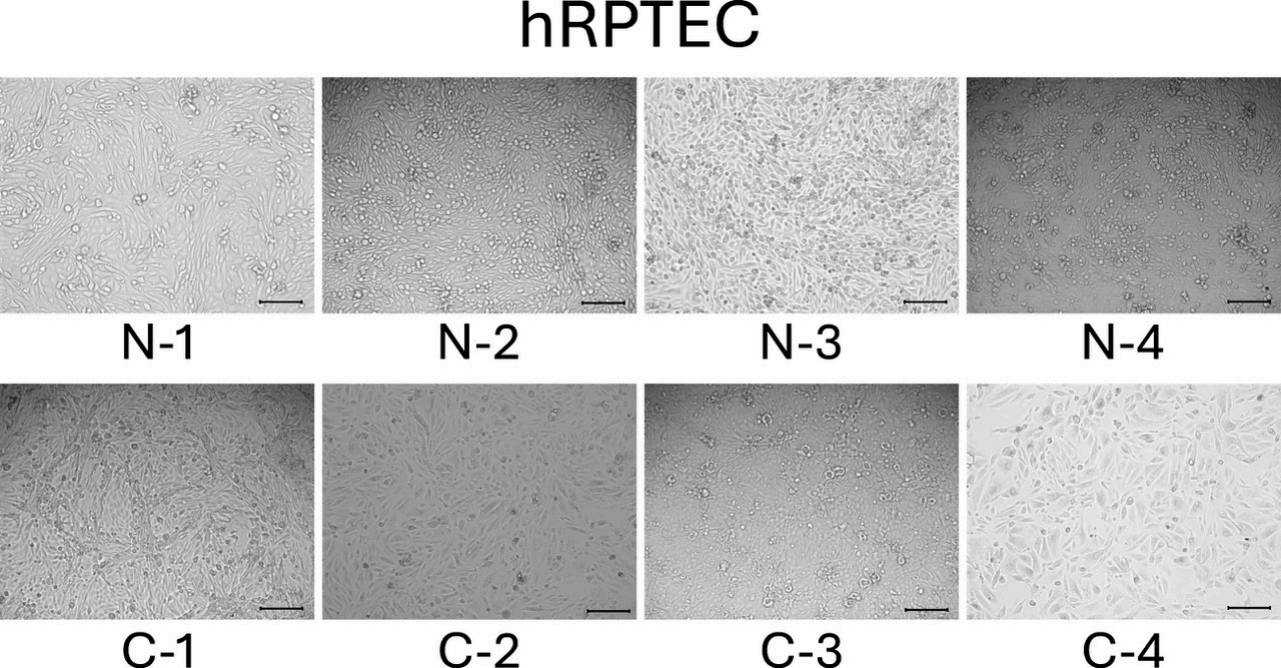
Primary hRPTECs from patients with mild CKD showed relatively elongated morphology. The top row shows cells from patients with normal kidney function (N-1–N-4), whereas the bottom row shows those from patients with mild CKD (C-1–C-4). Scale bar: 200 µm.

We observed the two-dimensional culture of all hRPTECs in passage 3 generation. All hRPTECs became confluent after approximately 1 week. Cell growth was slightly faster in normal-derived cells. hRPTECs from patients with normal kidney function showed a cobblestone appearance (Figure 1, the top row), whereas those from patients with mild CKD showed a relatively elongated morphology (Figure 1, the bottom row).

### Development of tubuloids from different patients simultaneously in a uniform condition

hRPTECs were then tri-dimensionalized and developed into tubuloids. We seeded hRPTECs on ultralow adherence plates with 5% FBS in Advanced RPMI media. After 2 days, we added Matrigel to bring its concentration to 10%. We defined day 0 as the day when Matrigel was added. On day 1, we added tubuloid media.

**Figure 2.**
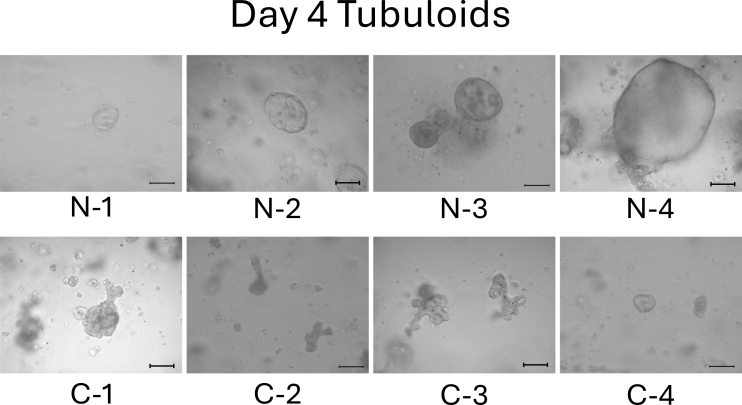
Morphology of normal cell- and mild CKD cell-derived tubuloids on day 4. The top row shows 3D-cultured cells from patients with normal kidney function (N-1–N-4), whereas the bottom row shows those from patients with mild CKD (C-1–C-4). Scale bar: 200 µm.

**Figure 3.**
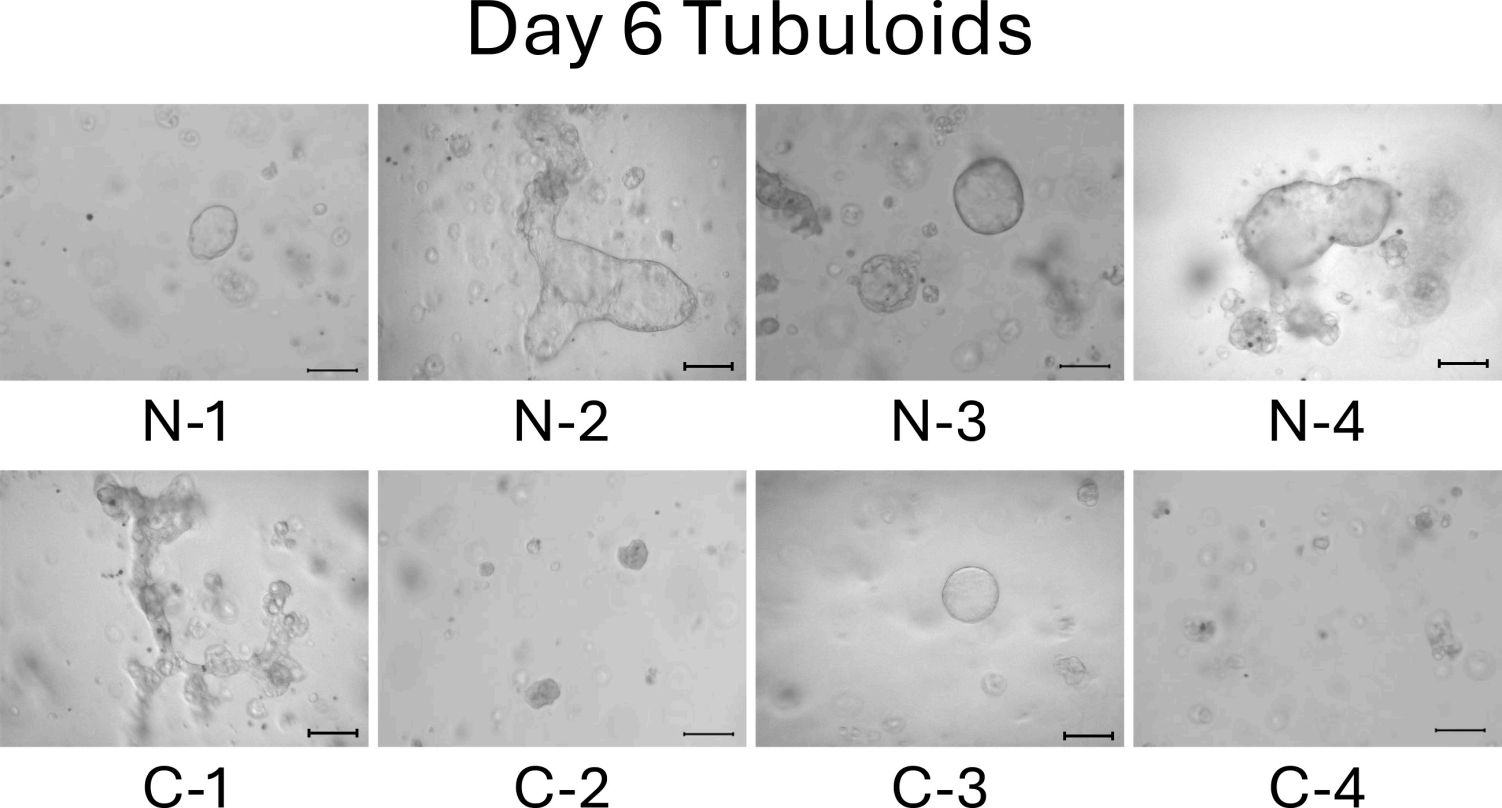
Morphology of normal cell- and mild CKD cell-derived tubuloids on day 6. The top row shows 3D-cultured cells from patients with normal kidney function (N-1–N-4), whereas the bottom row shows those from patients with mild CKD (C-1–C-4). Scale bar: 200 µm.

We observed the tubuloids on day 4. Normal cell-derived tubuloids formed a clear, round, and bubble-like structure (Figure 2, top). In contrast, mild CKD cell-derived tubuloids exhibited a bumpy and small structure (Figure 2, bottom). On day 6, the structure of the tubuloids was largely unchanged from those observed on day 4 (Figure 3).

**Figure 4.**
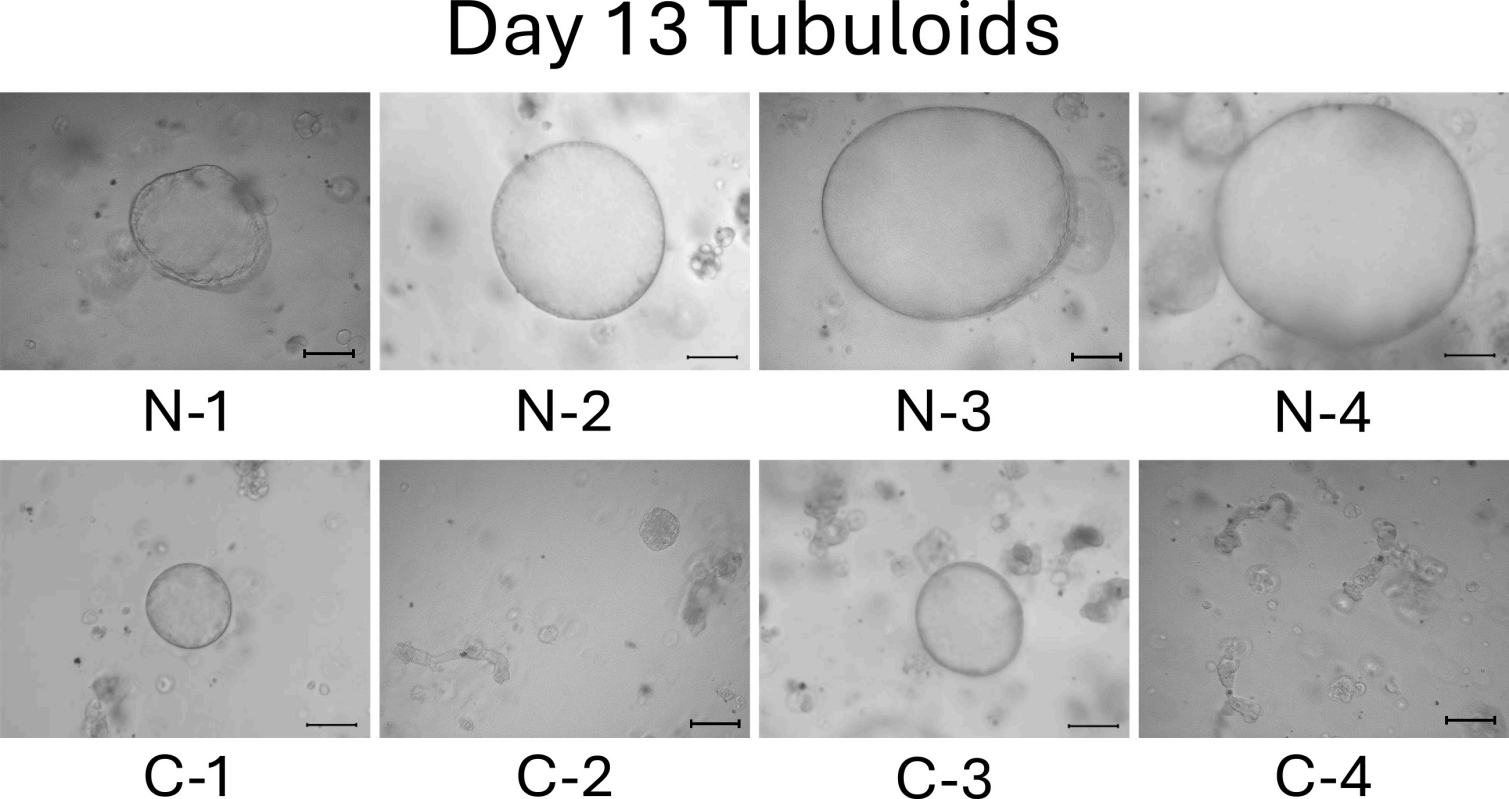
Morphology of normal cell- and mild CKD cell-derived tubuloids on day 13. The top row shows 3D-cultured cells from patients with normal kidney function (N-1–N-4), whereas the bottom row shows those from patients with mild CKD (C-1–C-4). Scale bar: 200 µm.

**Figure 5.**
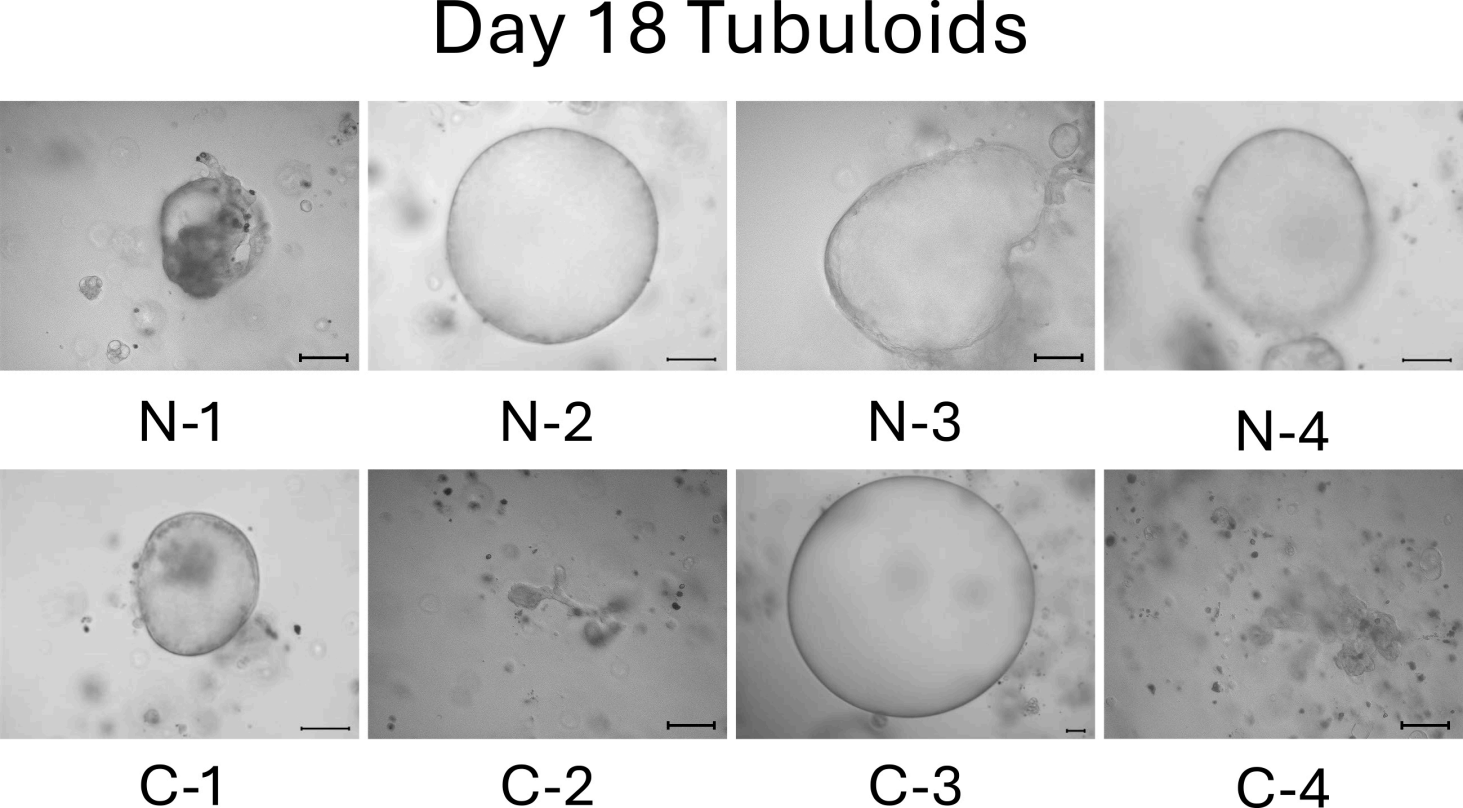
Morphology of normal cell- and mild CKD cell-derived tubuloids on day 18. The top row shows 3D-cultured cells from patients with normal kidney function (N-1–N-4), whereas the bottom row shows those from patients with mild CKD (C-1–C-4). Scale bar: 200 µm.

**Figure 6.**
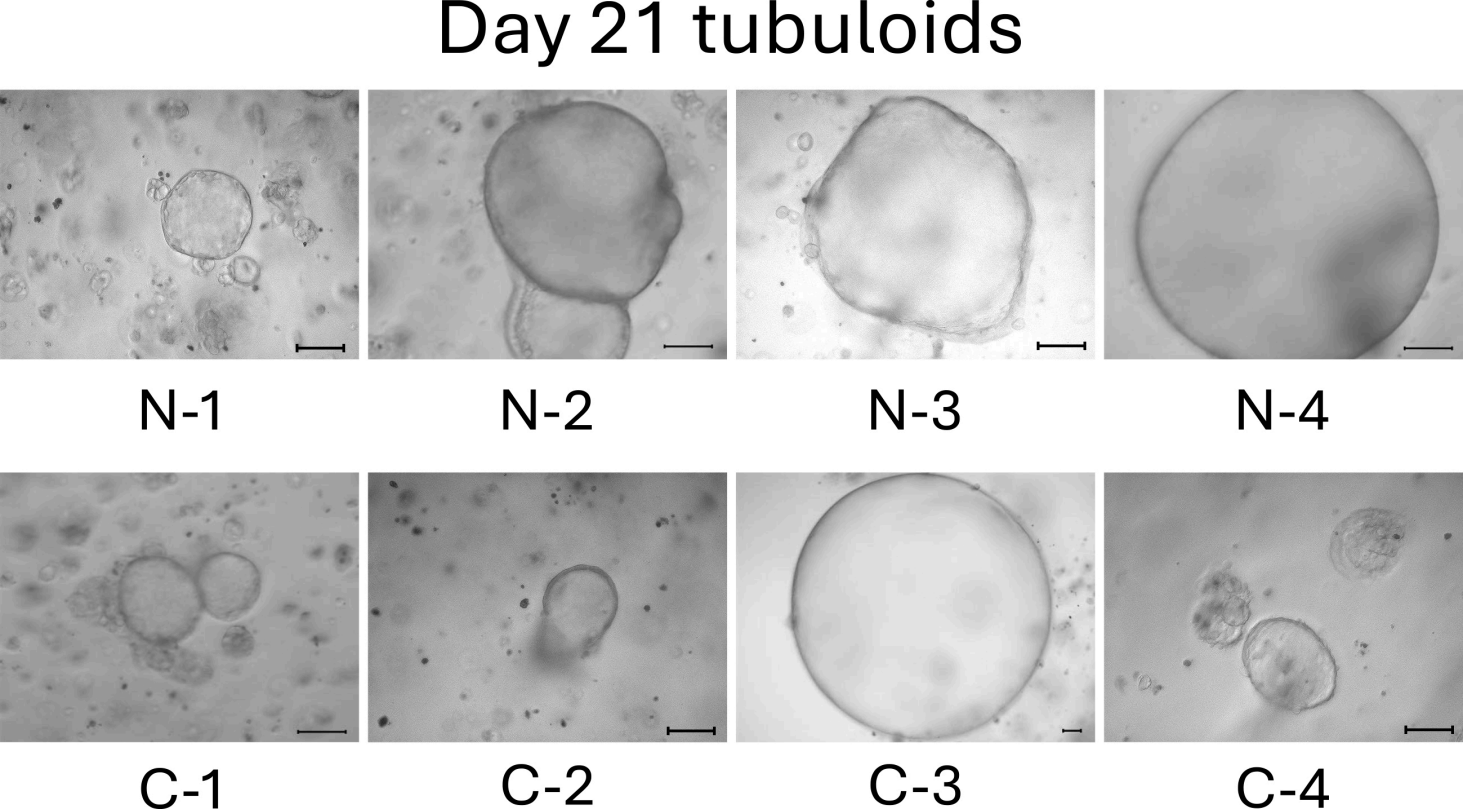
Morphology of normal cell- and mild CKD cell-derived tubuloids on day 21. The top row shows 3D-cultured cells from patients with normal kidney function (N-1–N-4), whereas the bottom row shows those from patients with mild CKD (C-1–C-4). Scale bar: 200 µm.

On day 13, the difference between normal cell- and mild CKD cell-derived tubuloids became more obvious. Normal cell-derived tubuloids exhibited large circular structures (Figure 4, top), whereas mild CKD cell-derived tubuloids exhibited sporadic and small circular structures (Figure 4, bottom). On day 18, the growth of tubuloids with circular structures was evident. Specifically, C-3 exceeded the size of the normal cell-derived tubuloids (Figure 5). On day 21, the C-2 and C-4 tubuloids, which had been difficult to transform into circular tubuloids, also exhibited a circular structure (Figure 6).

Before day 21, we observed that tubuloids derived from normal kidney cells grew faster than tubuloids derived from kidneys cells of patients with mild CKD. Meanwhile, tubuloids derived from kidneys cells of patients with mild CKD initially had a different growth pattern, which was eventually comparable to those of normal cell-derived tubuloids on day 21.

## Discussion

CKD is an age-related disease. However, most CKD models employ mice whose average lifespan is approximately 2 years, making it difficult to accurately represent the gradual progression of CKD over the course of a human lifespan. iPSC-derived kidney organoids also have difficulty in representing human aging and senescence because despite their complexity, they only reflect fetal kidneys.

Patient-derived organoids that we successfully developed are probably the ideal models for organ research as it not only represents the current pathology of the patient but also provides a true representation of aging and senescence, which is not possible with mice models or iPSC-derived organoids. A case study showed that renal tubular cysts shrink when tolvaptan is administered to an adult organoid model of autosomal dominant polycystic kidney disease (ADPKD); however, tolvaptan does not work in an iPSC-derived organoid model of ADPKD ^(13)^.

In addition, tubuloids can be formed by using a completely uniform protocol. For kidney organoids, a completely similar protocol for inducing the differentiation of iPSCs into NPCs cannot be employed ^(14)^. To compare the different genetic variants when using iPSC kidney organoids, we need to use the CRISPR/Cas9 system to produce the point mutation on the same iPSCs or embryonic stem cells ^(15), (16)^. Through this method, we can induce iPSC differentiation into NPCs and kidney organoids. Currently, comparing similar conditions between different individuals is not feasible with iPSC kidney organoids. However, tubuloids can be developed from multiple individuals using a similar protocol for comparison, although their developing phenotypes differ to some extent.

We have also succeeded in creating another CKD model by applying cisplatin to patient-derived tubuloids to accelerate cellular senescence ^(12)^. Thus, patient-derived tubuloids that accurately represent aging and senescence allow for observations that are not possible when using rodent models or iPSC organoids. Kidney tubuloids, which can be easily and rapidly produced in the same culture condition, can play an active role in elucidating various pathological conditions and developing novel drugs.

## Article Information

This article is based on the study, which received the Medical Research Encouragement Prize of The Japan Medical Association in 2023.

### Conflicts of Interest

None

### Sources of Funding

This work was supported by the Leading Initiative for Excellent Young Researchers from Ministry of Education, Culture, Sports, Science and Technology (to Y.M.), Grant-in-Aid for Research Activity Start-up from Japan Society for the Promotion of Science (to Y.M.), Innovation Idea Contest from Tokyo Medical and Dental University (TMDU) (in 2022 to Y.M. and in 2023 to Y.N.), Next-Generation Researcher Training Unit from TMDU (to Y.M.), Priority Research Areas Grant from TMDU (to Y.M.), Research Grant from Uehara Memorial Foundation (to Y.M.), Research Grant (lifestyle-related diseases) from MSD Life Science Foundation (to Y.M.), Medical Research Grant from Takeda Science Foundation (to Y.M.), and Academic Support from Bayer Yakuhin, Ltd. (to Y.M.).

### Acknowledgement

We thank the study participants who consented to the use of their resected kidneys.

### Author Contributions

M.M., Y.M., and Y.N. performed the experiments, collected and analyzed data, and wrote the manuscript. Y.M. and M.M. established the hRPTECs, and Y.N. and S.M. helped with the procedure. T.F., H.K., F.A., K.S., T.M., E.S., and S.U. supported the data analysis. Y.W., S.Y., and Y.F. resected the patients’ kidneys as standard treatment for malignant diseases. Y.M. developed the experimental strategy, supervised the project, and edited the manuscript. All authors discussed the results and implications and commented on the manuscript.

Makiko Mori and Yutaro Mori contributed equally to this work.

### Approval by Institutional Review Board (IRB)

IRB approval number: Ethics Committee of Tokyo Medical and Dental University (M2022-005)

### Disclaimer

Shinichi Uchida is one of the Associate Editors of JMA Journal and on the journal’s Editorial Staff. He was not involved in the editorial evaluation or decision to accept this article for publication at all.

### Data Availability Statement

Further information and requests for resources and reagents should be directed to and will be fulfilled by the Lead Contact, Yutaro Mori (y-mori.kid@tmd.ac.jp).
